# Endograft-preserving therapy of a patient with *Coxiella burnetii*-infected abdominal aortic aneurysm: a case report

**DOI:** 10.1186/1752-1947-5-565

**Published:** 2011-12-06

**Authors:** Geoffrey TL Kloppenburg, Eric DWM van de Pavoordt, Jean-Paul PM de Vries

**Affiliations:** 1 Department of Cardiothoracic Surgery, St. Antonius Hospital, Koekoekslaan 1, 3435 CM Nieuwegein, The Netherlands; 2 Department of Vascular Surgery, St. Antonius Hospital, Nieuwegein, The Netherlands

## Abstract

**Introduction:**

*Coxiella burnetii*, the causative agent of Q fever, may cause endocarditis and vascular infections that result in severe morbidity and mortality. We report a case of a *C. burnetii*-infected abdominal aorta and its management in a patient with a previous endovascular aortic aneurysm repair.

**Case presentation:**

A 62-year-old Caucasian man was admitted to our hospital three months after endovascular aortic aneurysm repair with a bifurcated stent graft. He had increasing abdominal complaints and general malaise. A computed tomography scan of his abdomen revealed several para-aneurysmal abscesses. Surgery was performed via midline laparotomy. The entire abdominal wall of his aneurysmal sac, including the abscesses, was removed. The vascular endoprosthesis showed no macroscopic signs of infection. The decision was made to leave the endograft in place because of the severe cardiopulmonary comorbidities, thereby avoiding suprarenal clamping and explantation of this device with venous reconstruction. The proximal and distal parts of the endograft were secured to the aortic wall and common iliac artery walls, respectively, to avoid future migration. Polymerase chain reaction for *C. burnetii *was positive in all specimens of aortic tissue. Specific antibiotic therapy was initiated. Our patient was discharged in good clinical condition after six days.

**Conclusions:**

In our patient, the infection was limited to the abdominal aneurysm wall, which was removed, leaving the endograft in place. Vascular surgeons should be familiar with this bailout procedure in high-risk patients.

## Introduction

*Coxiella burnetii*, the causative organism of Q fever, is increasingly reported to be associated with infections of abdominal aortic aneurysms and vascular grafts. *C. burnetii *is a small obligate intracellular Gram-negative bacterium related to the Rickettsiaceae family. Cattle, sheep, and goats are the primary reservoirs. Infection of humans usually occurs by inhalation of these organisms from air containing contaminated airborne barnyard dust. Symptoms of Q fever are polymorphic and non-specific and disease occurs in two stages: an acute stage that may present with headaches, chills, and respiratory symptoms and an insidious chronic stage. Acute Q fever is usually mild and recovery is spontaneous. However, *C. burnetii *is able to persist in host macrophages despite apparent cure, leaving patients, especially those with heart valve pathology or vascular defects, at risk of developing a chronic infection. Endocarditis is the main form of chronic Q fever (60% to 70% of all cases), followed by infections of aneurysms and vascular prostheses (9%) [1]. Multiple reports of cases of *C. burnetii *vascular infections have been published; overall mortality, most often due to vascular rupture, is 25% [2]. Given the significant morbidity and mortality of an infected aortic aneurysm or vascular endoprosthesis and the importance of targeted and prolonged antibiotic therapy besides surgery, the diagnosis of *C. burnetii *infection is crucial to a successful therapeutic outcome. We report a case of a *C. burnetii*-infected abdominal aorta in a patient with a previous endovascular aortic aneurysm repair.

## Case presentation

**Figure 1:**
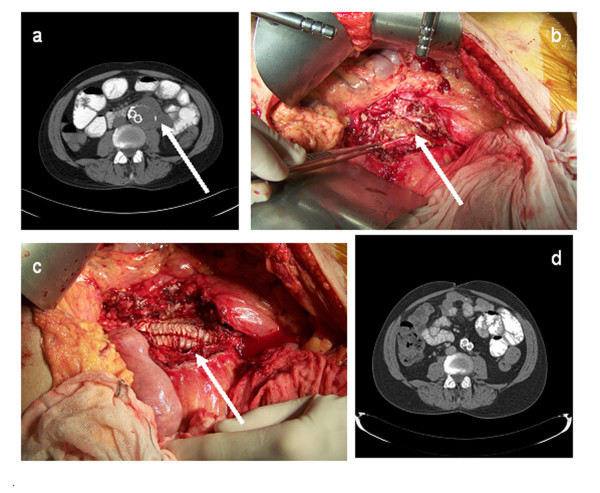
**(a) Para-aortic abscesses (arrow)**. (b,c) Peri-operative view of infected aneurysm wall (arrow). (d) A four-week post-operative computer tomography scan shows no abscesses.

A 62-year-old Caucasian man had a history of general malaise and recurrent fever. His general practitioner commissioned seven days of doxycycline therapy under the suspicion of Q fever, although the results of serological testing were negative. The patient still complained of general malaise and experienced progressive abdominal pain based on a symptomatic infrarenal aortic aneurysm of more than 6 cm, which was treated electively with an endovascular repair at another hospital. A bifurcated endograft was implanted without complications. The patient experienced a short episode of high fever early after the operation but remained free of symptoms for three months when he was readmitted to the hospital with increasing abdominal complaints and general malaise. On admission, he was afebrile. The results of a clinical examination were normal except for abdominal tenderness on deep palpation. Blood cultures were sterile in the absence of any recent antibiotic therapy. Laboratory results showed a white blood cell count of 11.5 × 10^9^/L and a C-reactive protein level of 24 mg/L. A serological test showed the persistence of *C. burnetii *phase II antibodies (immunoglobulin G [IgG]: 4096) and the appearance of phase I antibodies (IgG: 2048). A computed tomography (CT) scan of the abdomen revealed several para-aneurysmal fluid collections (Figure 1a).

At that point, the patient was referred to our hospital for surgical treatment of his infected abdominal aortic aneurysm. Surgery was performed via midline laparotomy. A visual inspection showed huge abscesses in his abdominal aortic wall. The entire abdominal wall of his aneurysmal sac, including the abscesses, was removed. The vascular endoprosthesis showed no macroscopic signs of infection, and because of the severe cardiopulmonary comorbidities, the decision was made to leave the endograft in place to avoid suprarenal clamping and explantation of this device with venous reconstruction. The proximal part and distal parts of the endograft were secured (with stitches) to the aortic wall and common iliac artery walls, respectively, to avoid future migration. The overlap zones of the endograft components were also secured with stitches. After that, the endograft was wrapped with omentum (Figure 1b,c). Blood, thrombus, and tissue cultures remained sterile, but polymerase chain reaction for *C. burnetii *was positive in all specimens, and specific staining of the aneurysmal sac for *C. burnetii *microorganisms was negative. Specific antibiotic therapy with Ciproxin (ciprofloxacin) 1500 mg/day and doxycycline 200 mg/day was initiated. Our patient was discharged in good clinical condition after six days.

Three months after surgery, he was readmitted to our hospital with subfebrile temperature, abdominal discomfort, nausea, incidental vomiting, and diarrhea. Blood cultures, transesophageal echocardiography, CT scans, and positron emission tomography scans were performed. The results of all of these measures were negative for reoccurrence of Q fever or infection of the endoprosthesis. Serological tests revealed no reoccurrence or persistence of *C. burnetii *infection. Antibiotic therapy on admittance included hydroxychloroquine, which is known to cause gastric complaints and general malaise. Our patient's complaints diminished after this was stopped and Ciproxin was restarted.

Six months after the first readmission, our patient was readmitted with cyanotic and painful toes to our hospital. Magnetic resonance imaging showed a functional endoprosthesis with no signs of stenosis or endoleak. Limited cutaneous systemic sclerosis in the absence of signs of macrovascular pathology was diagnosed. He was successfully treated with iloprost intravenously for 14 days, and an angiotensin-converting enzyme inhibitor and calcium antagonist were added to his medication. Our patient remained free of symptoms with unchanged and normal aspect of the endograft without signs of abscesses or infection, as seen on a CT scan (Figure 1d).

## Discussion

*C. burnetii *is the etiologic agent of Q fever, a zoonosis with a worldwide distribution in rural and, more recently, urban areas. Infection of humans usually occurs by inhalation of these organisms from air that contains airborne barnyard dust contaminated by dried placental material, birth fluids, and excreta of infected herd animals. Ingestion of contaminated milk, followed by regurgitation and inspiration of the contaminated food, is a less common mode of transmission. Our patient lived in an endemic region but had no known exposure to *C burnetii*.

Only about one half of all people infected with *C. burnetii *show signs of clinical illness. Acute cases of Q fever begin with a sudden onset of one or more of the following complaints: high fever (up to 104°F to 105°F), severe headache, general malaise, myalgia, confusion, sore throat, chills, sweats, non-productive cough, nausea, vomiting, diarrhea, abdominal pain, and chest pain. Fever usually lasts for one to two weeks. Weight loss can occur and persist for some time. Thirty to fifty percent of patients with a symptomatic infection will develop pneumonia. Additionally, a majority of patients have abnormal results on liver function tests and some will develop hepatitis. In general, most patients will recover, but 1% to 2% of infected people with acute Q fever die.

Chronic Q fever, characterized by infection that persists for more than six months, is uncommon but potentially lethal. Patients who have had acute Q fever may develop the chronic form within one to 20 years after initial infection. Endocarditis with negative blood cultures, the most frequent clinical presentation of chronic Q fever, accounts for 60% to 70% of the cases; next in frequency is vascular infection, which accounts for 8% of the cases [3]. Pre-existing cardiovascular disease may facilitate passage from acute to chronic Q fever despite antibiotic therapy. *C. Burnetii *as an obligate intracellular bacterium is able to persist in monocytes and macrophages that are present in aortic thrombus and damaged cardiac valves [4]. Transplant recipients, patients with cancer, and those with chronic kidney disease are also at risk of developing chronic Q fever. As many as 65% of patients with chronic Q fever may die of the disease.

Here, we report a case of *C. burnetii *infection of the wall of an abdominal aortic aneurysm in a patient with a previous endovascular aortic aneurysm repair. This unexpected diagnosis was made on the basis of serology and polymerase chain reaction on aspired fluid from an abdominal abscess. Recently, a report describing percutaneous management of a *C. Burnetii*-induced paravertebral abscess after emergency placement of an endograft was published [5]. *C. burnetii *aortic aneurysm infections are sometimes challenging to diagnose and treat. The cornerstone of treatment of aneurysms or infected vascular grafts is usually surgical excision in addition to antibiotic treatment [2]. Vascular reconstruction may include graft replacement or extra-anatomic bypass, which has a mortality rate of up to 33% in this patient group [6]. Alternatively, vascular reconstruction using autologus aortoiliac to femoral artery reconstruction with deep femoral-popliteal or superficial spiral saphenous veins can be performed. These procedures have good long-term patency rates but come with inherent morbidities such as compartment syndrome, deep venous thrombosis, and chronic limb swelling and a mortality risk of 7% to 15% [7,8]. In our patient, the abdominal aortic aneurysm wall itself, and not the endograft, was macroscopically infected. To avoid suprarenal clamping, explantation of the endograft, and complex reconstructive surgery, we chose to leave the endograft *in situ*. It is very important to secure the proximal and distal sealing zones to the native arteries as well as the endograft components to avoid endoleaks or migration during follow-up. Owing to the resection of the aneurysm wall, every type of endoleak will lead to life-threatening hemorrhages.

The diagnostic key is clinical awareness with a high index of suspicion. Q fever can be diagnosed serologically by high levels of specific antibodies to *C. burnetii*, immunohistology demonstrating *C. burnetii *within the cytoplasma of macrophages, or polymerase chain reaction-based methods. Cell culture isolation is not widely available as a diagnostic technique, as it requires a biosafety level 3 laboratory. Positive serology for *C. burnetii *is determined by the presence of phase I and II antibody titers. Acute Q fever is characterized by a predominance of phase II antibodies (more than 200), whereas chronic Q fever is associated with high levels of phase I antibodies (greater than or equal to phase II titers) [3].

There are no specific guidelines for Q fever vascular prosthesis infections; experience has been derived from the treatment of Q fever endocarditis. Oral antibiotic regimens include doxycycline and hydroxychloroquine or doxycycline and a quinolone. The duration of antibiotic treatment is best targeted on serological monitoring of phase I antibody titers. A decrease of phase I IgG and IgA antibody titers to below 200 is a criterion for clinical cure. Serological evaluation is recommended monthly for the first six months after antibiotics are discontinued and every three months for two years before the patient can be considered cured [9].

## Conclusions

Chronic *C. burnetii *aortic abdominal wall infection is a life-threatening disease with devastating complications and a poor prognosis. In our patient, the infection was limited to the abdominal aneurysm wall and the endograft could be left in place. Vascular surgeons should be familiar with this bailout procedure in high-risk patients. The first episode of Q fever may remain asymptomatic, making an early and accurate diagnosis fairly difficult. We recommend systematic serological testing for *C. burnetii *in all patients with the combination of possible exposure, an abdominal aortic aneurysm and unexplained fever, abdominal pain, or weight loss.

## Abbreviations

CT: computed tomography; IgG: immunoglobulin G.

## Consent

Written informed consent was obtained from the patient for publication of this case report and any accompanying images. A copy of the written consent is available for review by the Editor-in-Chief of this journal.

## Competing interests

The authors declare that they have no competing interests.

## Authors' contributions

GK analyzed and interpreted the patient data and, together with J-PV, was responsible for drafting the manuscript. EP was the main surgeon and was responsible for critically revising the manuscript. All authors read and approved the final manuscript.
